# All roads lead to NF-κB: the NF-κB pathway as a major target for intestinal inflammatory disorders

**DOI:** 10.3389/fimmu.2026.1816653

**Published:** 2026-04-30

**Authors:** Rasul Khasanov, Michael Boettcher, Lucas M. Wessel, Karl-Herbert Schäfer, María Ángeles Tapia-Laliena

**Affiliations:** 1 Department of Pediatric Surgery, Medical Faculty of Mannheim, University of Heidelberg, Mannheim, Germany; 2 Working Group Enteric Nervous Systems (AGENS), University of Applied Sciences Kaiserslautern, Kaiserslautern, Germany

**Keywords:** Crohn’s disease (CD), gut-brain axis, inflammatory bowel disease (IBD), microbiota dysbiosis, necrotizing enterocolitis (NEC), NF-κB-induced neuroinflammation, neutrophil extracellular traps (NETs), ulcerative colitis (UC)

## Abstract

This review aims to comprehensively examine the role of the NF-κB signalling pathway as a central mediator of intestinal inflammation, integrating evidence from microbiota dysbiosis, immune activation, neutrophil extracellular trap (NET) formation, and the gut–brain axis, and to evaluate current and emerging NF-κB-targeted therapeutic strategies for inflammatory intestinal disorders. The NF-κB family comprises transcription factors that control key processes in immune responses and inflammation by regulating specific gene expression. NF-κB signalling mediates intestinal inflammatory responses at different levels including cytokine secretion, inflammasome signalling, the recruiting of immune cells and antibody production. The NF-κB pathway acts as sensor of microbiota changes and is strongly activated by bacterial toxins such as LPS, MDP or TMAO. Thus, microbial dysbiosis activates pro-inflammatory NF-κB, producing epithelial barrier dysfunction that can lead to a “leaky gut” syndrome, allowing pro-inflammatory factors to leak into the systemic circulation. Consequently, the inflammation originating in the intestine spreads to other organs like the brain, where it might contribute to the development of neurodegenerative disorders, such as Parkinson’s disease. In addition, NF-κB also promotes intestinal inflammation in response to Neutrophil Extracellular Traps (NETs) formation, promoting tissue damage and lesions of the intestinal epithelial lining. Therefore, a dysregulated NF-κB signalling appears often in multiple chronic intestinal inflammatory conditions, such as Crohn’s Disease (CD) and Ulcerative Colitis (UC), Celiac Disease (CeD) or microscopic colitis. In addition, NF-κB is strongly activated in Irritable Bowel Syndrome (IBS) and in acute intestinal inflammation such as Necrotizing Enterocolitis (NEC). Confirming this evidence, inhibition of the NF-κB pathway by drugs, peptides or natural compounds has been demonstrated to ameliorate the symptoms in many of these inflammatory diseases. In this review, we explore the role of the NF-κB pathway in intestinal inflammation, given its essential role in linking microbiota dysbiosis, infections and chronic inflammation. Finally, we propose the NF-κB pathway as a main therapeutic target for inflammatory intestinal disorders and discuss current inhibitory therapies in use.

## 1 Introduction

### 1.1 The NF-κB pathway

The nuclear factor-κB (NF-κB) pathway is a family of transcription factors that regulate the expression of many genes that participate in main cellular and physiological processes, such as: cell cycle, cell survival, apoptosis, cell growth, cell adhesion, cell migration, angiogenesis, inflammation and immune responses (1, 2). Therefore, the NF-κB pathway is closely related to many pathologies, especially to inflammatory conditions (1, 3).

The NF-κB family is composed by five main subunits: p65/RelA, RelB, c-Rel, p50/p105 (NF-κB1) and p52/p100 (NF-κB2), which form homo- and/or heterodimers in the cytoplasm, where they are bound to the inhibitory IκB proteins (IκB-α, IκB-β or IκB-ϵ). The IκBs mask the nuclear localization signal (NLS) of the dimers, keeping them inactive in the cytoplasm (4, 5).

**Figure 1:**
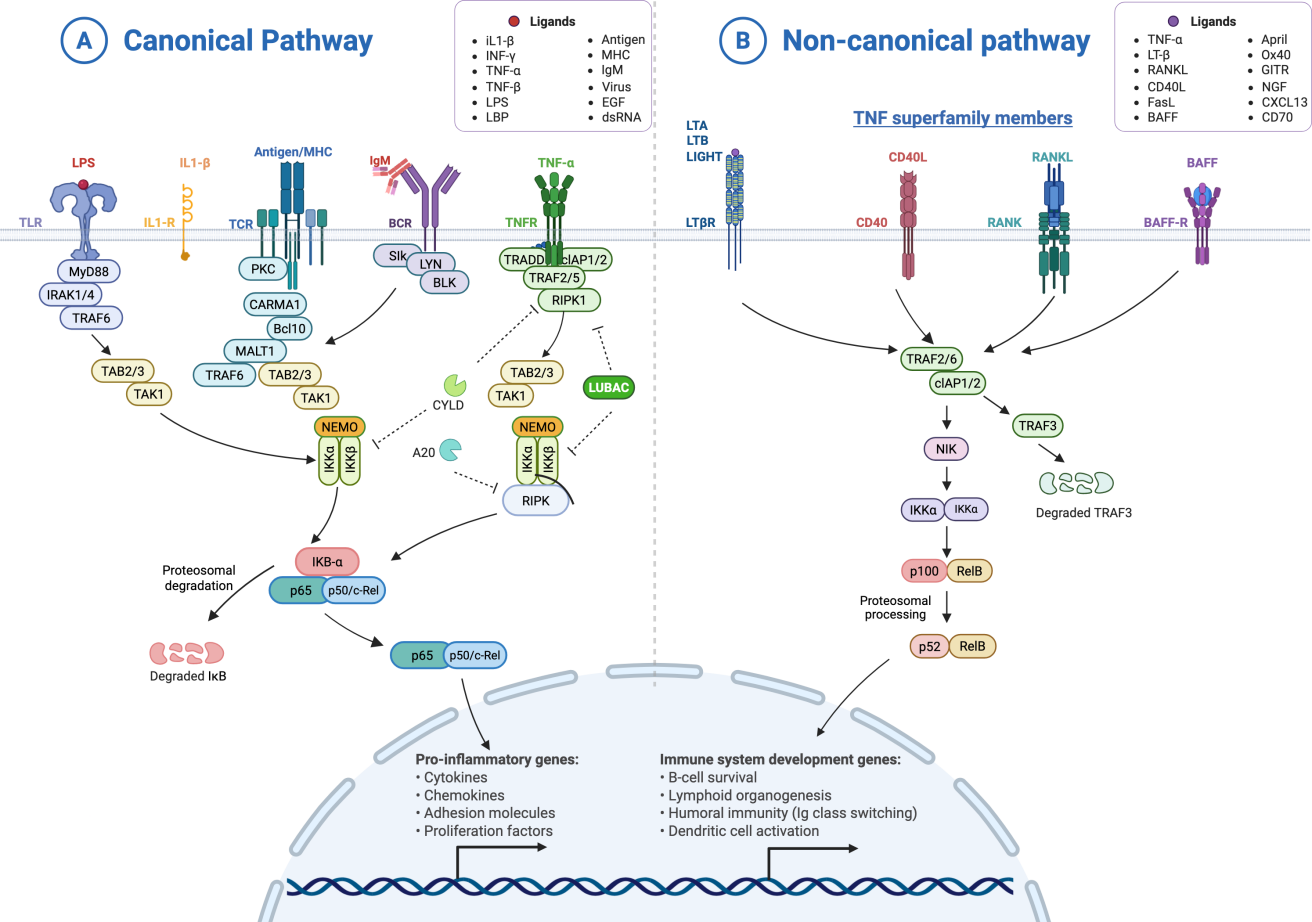
Schematic illustration of the canonical (classical) and non-canonical (alternative) NF-κB activation pathways. **(A)** In the canonical pathway, stimulation by TNFα, IL-1β, or LPS activates the IKK complex (IKKα/IKKβ/NEMO), leading to IκBα phosphorylation, ubiquitination, and proteasomal degradation, releasing p65/p50 dimers for nuclear translocation and transcription of pro-inflammatory target genes including cytokines, iNOS, COX-2, and NLRP3. **(B)** In the non-canonical pathway, TNFR superfamily ligands (BAFF, CD40L, LTβR, RANKL) stabilise NIK through TRAF3 degradation, activating IKKα homodimers that process p100 to p52, enabling p52/RelB nuclear translocation and transcription of genes involved in B-cell survival, lymphoid organogenesis, and dendritic cell activation (created with BioRender).

There are two fundamental NF-κB activation pathways (see **Figure 1**): A first pathway is termed classical or canonical, commonly comprised by p65-p50 dimers but also others involving c-Rel, where NF-κB activation depends on the IKK complex activity and IκB degradation. Upon stimulation by proinflammatory cytokines, the signals converge on the IKK complex, formed by three subunits—IKKα and IKKβ and IKKγ/NEMO. Then, IKKβ phosphorylates the inhibitory IκBs, which are degraded by the proteasome, allowing the NF-κB dimers to translocate to the nucleus, where they inhibit or activate the transcription of their target genes (4, 5). As many of these genes are antimicrobial peptides, cytokines, chemokines, growth factors, stress response proteins, and anti-apoptotic proteins (6), the canonical NF-κB pathway is principally related to inflammation, cell proliferation and survival signals (1, 2).

The second pathway, named non-canonical or alternative, is characterized by the presence of p100-RelB heterodimers, in which p100 acts as the inhibitory subunit. In this case, the activation signal, mainly of the TNFR receptors family, converges on the protein kinase NIK, which activates dimers of IKKα. Phosphorylation of p100 by IKKα promotes its cleavage, releasing the fragment containing ankyrin repeats. The resulting p52-RelB dimer translocates to the nucleus, where it activates genes involved in humoral immunity and cell differentiation (4, 5). Thus, the non-canonical NF-κB pathway is important for antibody production, lymphoid organ development, lymphocyte survival, and dendritic cell activation (2). When dysregulated, it may lead to ulcerative colitis and lymphoid malignancies (6).

Additionally, there can be a third activation route, the NF-κB pathway can also be induced in response to DNA damage to promote cell survival. The cascade signal of DNA Damage Response factors ends up activating NF-κB, which starts the transcription of pro-survival genes, leading to evasion of apoptosis, but facilitating later tumour survival and resistance to both chemotherapy and radiotherapy in cancer processes (7).

Regarding inflammation, it is mainly initiated by NF-κB through the transcriptional regulation of genes involved in immune activation and cell survival, including cytokines, chemokines, and growth factors.

During inflammation, the NF-κB signalling is hyperactivated, leading to the abundant expression of inflammation-associated genes (4, 6). In addition, the NF-κB pathway interacts up- and downstream with several important cellular pathways, including PI3K/AKT, MAPK, JAK-STAT, TGF-β, Wnt, Notch, Hedgehog, and TLR, which increases its number of possible inducers as well as its effects exponentially (2, 6).

This process is amplified by immune cells such as macrophages and dendritic cells that produce additional pro-inflammatory cytokines including interferon gamma (IFNγ), interleukin-1 beta (IL-1β), and tumour necrosis factor-alpha (TNFα), which in turn reactivate the pro-inflammatory NF-κB pathway, generating an exponential loop (8, 9).

Innate immune cells (macrophages, dendritic cells, and neutrophils) further activate NF-κB through their Pattern Recognition Receptors (PRRs): Toll-like receptors (TLRs), RIG-I-like receptors, NOD-like receptors, and C-type lectin receptors (10). They detect pathogen-associated molecular patterns (PAMPs) of microbial and damage-associated molecular patterns (DAMPs) released by necrotic cells or damaged tissues (6, 10). Thus, in situations of injury or infections, the immune cells PRRs act as sensors that activate the canonical NF-κB pathway, which induces the transcription and secretion of more pro-inflammatory cytokines together with the NOD-, LRR- and pyrin domain-containing protein 3 (NLRP3) inflammasome. This activation leads to a signalling that directly contributes to inflammation or indirectly promotes the differentiation of inflammatory T cells mediators. The latter coordinate the elimination of pathogens and infected or damaged cells (6, 10). Mechanistically, the NF-κB–NLRP3 axis operates through a two-step activation model: first, NF-κB-dependent transcription provides the ‘priming’ signal by upregulating the expression of NLRP3, pro-IL-1β, and pro-IL-18; second, a diverse range of danger signals—including ATP, reactive oxygen species (ROS), potassium efflux, and bacterial toxins—triggers NLRP3 inflammasome assembly, leading to caspase-1 activation, maturation and secretion of IL-1β and IL-18, and Gasdermin D-mediated pyroptosis. In the intestinal mucosa, continuous microbial exposure provides a persistent priming signal, lowering the threshold for inflammasome activation in response to additional triggers such as epithelial barrier breach, pathogen invasion, or microplastic or similar toxic exposures (6, 10).

Moreover, if the immune cells are infected or damaged, they activate NF-κB thus initiating the synthesis of pro-inflammatory cytokines, such as TNFα, IL-1β, and interleukin-6 (IL-6), and so starting inflammation and recruiting other immune cells to combat pathogens or repair damaged tissues (6).

Given that the NF-κB family coordinates important immune and inflammatory processes, such as the secretion of pro-inflammatory cytokines, T and B cell development, activation, and function, antibody production and immunological memory formation, it is a crucial component of both innate and adaptive immunity, and plays a vital role in immune response regulation. In this review, we aim to provide a comprehensive analysis of NF-κB-mediated intestinal inflammation, covering its activation by microbiota dysbiosis, its bidirectional interplay with neutrophil extracellular traps (NETs), and its role across the spectrum of inflammatory intestinal disorders—from functional conditions such as IBS to life-threatening neonatal emergencies such as NEC. We further evaluate current pharmacological and natural compound-based strategies for therapeutic NF-κB inhibition in these conditions.

### 1.2 The NF-κB pathway mediates intestinal inflammation

NF-κB signalling orchestrates innate and adaptive immune responses and the production of immunoregulatory proteins, anti-inflammatory cytokines, antimicrobial peptides, and other tolerogenic factors in the intestine. Furthermore, genetic studies have revealed critical cell type-specific roles for NF-κB proteins in intestinal immune homeostasis, inflammation, and restitution that contribute to the etiopathology of Inflammatory Bowel Disease (IBD)-associated manifestations (11).

Importantly, intestinal epithelial cells (IECs) express different PRRs like TLRs (including TLR2, TLR3, TLR4 and TLR5) mainly on their basolateral and apical cell membranes. This expression configuration helps to guarantee that only invasive bacteria, but not luminal bacteria, activate TLRs signalling in the intestinal mucosa. Upon microbial ligands, these receptors induce signalling cascades that end in the activation of pro-inflammatory NF-κB (6, 12). Moreover, in response to damage or stress, epithelial cells release cytokines, including IL-25, IL-33 and IL-1α, to activate local innate lymphoid cells (ILCs) and memory lymphocytes (12). Accordingly, a high expression of the pro-inflammatory cytokine interleukin-36 (IL-36), has been identified in Ulcerative Colitis (UC) as well as in the colon of Crohn’s Disease (CD) patients (13).

The intestinal mucosa is maintained by the Wnt/β-catenin signalling pathway, which interacts reciprocally with the NF-κB pathway in a cell type and stimulus-dependent manner. In the context of intestinal pathobiology, usually Wnt activation inhibits NF-κB signalling mainly through direct interaction of β-catenin with NF-κB p65 and p50 dimers, which inhibits their DNA binding and target gene expression in colonic epithelial cells (14).

However, also NF-κB signalling interferes back on the Wnt pathway: in IECs cell lines, upon proinflammatory cytokines, IKK-α phosphorylates β-catenin to drive the expression of select TCF target genes. Consistently, activation of NF-κB signalling enhances β-catenin/TCF-dependent gene transcription, and promotes the dedifferentiation of intestinal epithelial cells. An increase of stem cell markers in UC samples suggests that this NF-κB/β-catenin interaction also occurs during intestinal inflammation (14).

Concerning the Enteric Nervous System (ENS), we established that Wnt signalling exerts an anti-inflammatory activity by inhibiting NF-κB in rat primary ENS cultures, as proved by the downregulation of pro-inflammatory cytokines expression (15, 16).

The ENS is composed of neurons and enteric glia cells (EGC), which also play an important role to keep intestinal homeostasis and mucosal integrity. We found that in response to pro-inflammatory cytokines, EGC secrete Neurotrophin nerve growth factor (NGF), which can increase visceral sensitivity and, on the other hand, appears to improve gut inflammation (17).

Usually, patients of CD and Necrotizing Enterocolitis (NEC) present pathological changes in EGC. The loss of populations of enteric glial fibrillary acidic protein (GFAP) positive (+) glia cells is associated to a haemorrhagic jejunoileitis. We observed that pro-inflammatory cytokines like IL-1β, TNF-α, and lipopolysaccharides (LPS) promote a high increase in GFAP+ enteric glia, which might in turn be involved in modulating the integrity of the bowel during inflammation (18).

Recently, we also have demonstrated that the NF-κB pathway and other NF-κB-related and pro-inflammatory factors participate in the inflammation in Hirschsprung’s disease (19), which is characterized by a deficient intestinal innervation by the ENS.

Hence, the NF-κB pathway coordinates intestinal inflammation through complex processes, in which immune cells, epithelial cells and neuronal cells are involved and work together to maintain intestinal integrity and homeostasis.

### 1.3 Microbiota dysbiosis activates pro-inflammatory NF-κB signalling

The intestinal microbiota is essential in maintaining immune homeostasis, influencing both innate and adaptive immune responses (20). Certainly, alterations in the gut microbiome, called dysbiosis, in combination with the individual genetics can transform the immune system, the ENS and the central nervous system, and so impair physiological barriers. Therefore, they contribute to various disorders such as IBS, IBD or neurodegeneration and mental issues (21, 22), but also to autoimmune diseases (20).

The NF-κB pathway acts as an immunological gatekeeper in the connections between microbial dysregulation and aberrant immune activation, and microbial metabolites or changes in microbial composition can alter NF-κB signalling and so lead to chronic inflammation and autoimmunity conditions (20). Additionally, bacterial fermentation products, including short-chain fatty acids (SCFAs), also show immunomodulatory effects by influencing T-cell differentiation and cytokine profiles (20).

Consequently, therapeutic approaches targeting microbial restoration, such as precision probiotics, faeces transplantation, and nutritional interventions, can be a therapeutic option to restore immune balance in the future (20).

Lipopolysaccharides (LPS) are microbiome-derived glycolipids considered among the most potent pro-inflammatory neurotoxins known. In the gastrointestinal (GI)-tract, anaerobic Gram-negative bacteria form the microflora, including *Bacteroides fragilis* and *Escherichia coli*, are responsible for the major sources of LPS in humans (23).

Microbiome-generated LPS and other endotoxins are able to increase intestinal permeability, cross GI-tract biophysiological barriers into the systemic circulation and also through the blood-brain barrier (BBB) into the brain, a pathological process known as ‘leaky gut syndrome’, which amplifies during aging and in vascular disorders (23).

NF-κB stimulation by LPS has been appointed as the molecular link between the intestinal microbiome and the brain inflammation. This ‘leaky gut syndrome’ connecting the gut-brain axis starts as follows: LPS, mainly secreted by Gram-negative enterobacteria, reaches the brain from systemic circulation after crossing first the GI-tract barrier and later the BBB. In brain tissue, LPS activates pro-inflammatory NF-κB dimers (p50/p65), which start the transcription of different NF-κB-sensitive microRNAs, including miRNA-30b, miRNA-34a, miRNA-146a and miRNA-155 (23, 24). These miRNAs will later downregulate their mRNA targets, such as complement factor H (CFH), a key repressor of the innate-immune response. Down-regulated CFH expression activates the complement-system and promotes neuro-inflammation. Another important target is the mRNA encoding the neuron-specific neurofilament light (NF-L) chain protein. While NF-L has been reported to be up-regulated in Alzheimer’s disease (AD) and other inflammatory neurodegenerative disorders, NF-L is significantly down-regulated within neocortical neurons, and this may account for neuronal atrophy, loss of axonal caliber and alterations in neuronal cell shape, modified synaptic architecture and network deficits in neuronal signalling capacity (23).

Actually, this may be the circuit used by Gram-negative anaerobic bacterial species already related to the onset of AD (24). Moreover, there is additional evidence by the high amounts of LPS detected in aged human brain, and even higher levels found in AD (23). Thus, the network gut-microbiota-derived LPS-NF-κB-miRNA-neuroinflammation signalling underscores a direct pathological link between the LPS of GI-tract microbes and the inflammatory neurodegeneration (23).

In addition to LPS, altered enteric microbiota produce trimethylamine (TMA) from dietary substrates such as choline, L-carnitine, and betaine (found in red meat, eggs, fish, and dairy), which is subsequently oxidised to trimethylamine-N-oxide (TMAO) by hepatic flavin-containing monooxygenases (FMO3) in the host liver, which also crosses the BBB and activates pro-inflammatory NF-κB signalling, exacerbating neuroinflammation further (25). Moreover, TMAO induces amyloid-beta and tau aggregation (tau being a microtubule-associated protein whose hyperphosphorylation and aggregation into neurofibrillary tangles is a hallmark of Alzheimer’s disease pathology), disrupts the integrity of the BBB, and promotes neuronal death. Again, this reveals a critical link between neurodegeneration and the gut microbiome, via the gut-brain communication axis. Thus, neurodegenerative diseases, such as AD and Parkinson’s disease (PD), are severe age-related disorders with complex and multifactorial causes (25).

Indeed, there is increasing evidence that PD might start in the gut, thus involving and compromising also the enteric nervous system (ENS). We have showed that Parkinson mice models present functional and molecular changes in the gut long before motoric disease onset (26), as well as that bacterial amyloids induce the secretion of pro-inflammatory cytokines by enteric glia, DNA damage and replication, which triggered immune cells *in vivo* (27). Moreover, we have detected increased levels of intestinal inflammation and permeability markers in faeces of PD patients, confirming intestinal inflammation as contributing factor in the pathogenesis of PD (28).

A similar regulatory mechanism of the gut flora inducing acute lung injury has been described. Again, gut microbiota imbalance induced LPS may alter the TLR4/NF-κB signalling pathway in the lung immune system, activating inflammation and oxidative stress in the lung and mediating lung injury through the regulation of the gut barrier. Confirming this, faecal microbiota transplantation (FMT) impaired the activity of the TLR4/NF-κB signalling pathway in the lung and decreased oxidative stress in animals with acute lung injury by restoring the gut microecology (29).

On the contrary, bacterial LPS may exert a protective effect on the ENS in the intestine. The ENS has to adapt to microenvironmental changes within the gut and is therefore dependent on a neural stem cell niche to keep the ENS functional throughout life. We observed that LPS enhances the proliferation of enteric neural stem/progenitor cells NSPCs in a dose-dependent manner. In the case of inflammatory disease or trauma where the liberation and exposure to LPS will be increased, the expansion of NSPCs could be a first step towards regeneration of the ENS. The reduced and altered differentiation, as well as the induction of cytokine signalling, demonstrates that the stem cell niche may take part in the LPS-transmitted inflammatory processes in a direct and defined way (30).

Furthermore, NF-κB dysregulation also stands out as necessary for intestinal permeability, which, for instance, characterizes IBS with predominant diarrhoea (IBS-D). NF-κB was proved to play a key role in inflammatory response of intestine and resultant disruption of tight junction integrity, whose activity could be inhibited by TNF Receptor-Associated Factor 3 (TRAF3). Another study using miR-29 knockout mice and patient samples, found again that TRAF3 regulates the NF-κB-MLCK signalling pathway and is involved in the pathogenesis of intestinal hyperpermeability in IBS-D (31).

Microplastics (MPs) have recently emerged as a new cause for intestinal inflammation and microbiota dysbiosis, since they been found to accumulate in the digestive system, where they can damage the gut barrier (32–34). High quantities of MPs have been detected in seafood (35, 36), fruits and vegetables (37), salt, sugar, or tap and bottled water (38). They can also pass into the food from packaging, kitchen utensils or plastic bottles (39, 40). Hence, MPs abundance in the food chain has already been proved by several studies (41–43) and also evidenced by its isolation in human stool samples (44). When MPs enter the gastrointestinal tract, they harm it in various ways, such as microbiota disturbances, mutagenicity, cytotoxicity, reproductive toxicity, neurotoxicity, and increasing oxidative stress (45, 46). As a result, exposure to MPs induces gut microbiota dysbiosis, intestinal barrier dysfunction and metabolic perturbations (47–49). The resulting microbiota composition is partially dominated by *Firmicutes* and *Bacteroidetes* (49), which represent an important LPS source.

Importantly, current evidence indicates that microplastics activate intestinal NF-κB through both direct and microbiota-mediated mechanisms. Direct activation occurs when microplastics physically interact with intestinal epithelial cells, inducing oxidative stress and TLR4-dependent NF-κB signalling independently of microbiota changes. Concurrently, microplastics cause microbiota dysbiosis enriching LPS-producing Gram-negative bacteria, providing an additional indirect route to NF-κB activation. These mechanisms likely operate in parallel, with relative contributions depending on particle size, polymer type, and exposure duration. It should be noted that NF-κB/NLRP3 pathway activation is not unique to microplastics but is a shared response to multiple stimuli discussed in this review, including LPS, MDP, and TMAO; microplastics thus represent one converging activator among many.

Accordingly, the NF-κB pathway has been identified as mediator of the intestinal inflammatory effects of MPs by different researchers. Polystyrene nanoplastics (PS-NPs) activated inflammatory NF-κB/NLRP3 pathways, induced the expression of intestinal IL-1β and IL-18, which triggered macrophages and neutrophils recruitment. In addition, PS-NPs decreased the levels of intestinal tight junction proteins (Claudin-1, Occludin, and ZO-1), resulting in an increase in intestinal permeability and elevated LPS levels that further activated TLR4/NF-κB/NLRP3/Gasdermin D (GSDMD) pathways in the liver, inducing liver inflammation and hepatocyte pyroptosis. The impairment of liver function was positively correlated with intestinal inflammation and barrier disruption (50). Further *in vivo* experiments on Bisphenol P (BPP) showed similar results: BPP caused gut microbiota dysbiosis, activated the LPS/TLR4/NF-κB signalling pathway, triggered an inflammatory response, increased intestinal permeability, and promoted bacterial translocation leading to intestinal barrier disruption (51).

Finally, the gastrointestinal microbiome, age-related dysbiosis induced chronic inflammation by NF-κB may also be boosting dysregulation of the immune function that occurs with aging. Aging is associated with persistent activation of the immune system, witnessed by a high circulating level of inflammatory markers and activation of immune cells in the circulation and in tissue, a condition called “inflammaging”. Considering that inflammation can be reduced through diet and exercise, changes in lifestyle may increase the health span (52).

Altogether, microbiota dysbiosis induces pro-inflammatory NF-κB signalling, which increases intestinal permeability, producing the “leaky gut” syndrome. This status allows many pro-inflammatory cytokines and factors to reach the systemic circulation and other organs such as the brain; where they subsequently promote inflammation and contribute to the progression of neurodegeneration disorders.

### 1.4 Neutrophil extracellular traps activate pro-inflammatory NF-κB

IBD is usually characterised by chronic non-resolving gut mucosal inflammation involving innate and adaptive immune responses. Neutrophils, as first responders of the innate immune system, contribute to IBD through multiple mechanisms, including the release of reactive oxygen species (ROS) and pro-inflammatory cytokines and the recruitment of further immune cells. In addition, they induce the formation of neutrophil extracellular traps (NETs): these are extracellular webs of chromatin, microbicidal proteins and oxidative enzymes that are released by neutrophils in order to contain pathogens (53, 54). Since NETs act as a defence mechanism at the intestine, whose mucosa is continuously exposed to high levels of bacteria, viruses and fungi, in IBD conditions there is an increment in NET formation to combat these microbial threats and promote tissue repair; however, excessive NETs can lead to epithelial injury, barrier disruption, microbial imbalance, and thrombotic risk. Therefore, NETs can also negatively potentiate and perpetuate gut inflammation, so they may contribute to IBD-related complications (53, 54).

Importantly, NF-κB and NETs engage in a bidirectional amplification loop that is central to intestinal immunopathology. On one hand, NF-κB activation in neutrophils is a prerequisite for NETosis: NF-κB-dependent transcription of pro-inflammatory genes primes neutrophils for NET release, and pharmacological NF-κB inhibition consistently reduces NET formation across multiple experimental models (55, 56). On the other hand, once released, NETs themselves act as potent NF-κB activators in surrounding cells. NET-associated components, including histones, high-mobility group box 1 (HMGB1), and neutrophil elastase, serve as DAMPs that engage TLR2, TLR4, and RAGE receptors on epithelial cells and macrophages, thereby triggering canonical NF-κB signalling and perpetuating cytokine release (53, 54). This self-sustaining circuit explains why NET-driven inflammation is particularly difficult to resolve in the intestinal mucosa, where continuous microbial exposure provides an ongoing stimulus for neutrophil recruitment.

**Figure 2:**
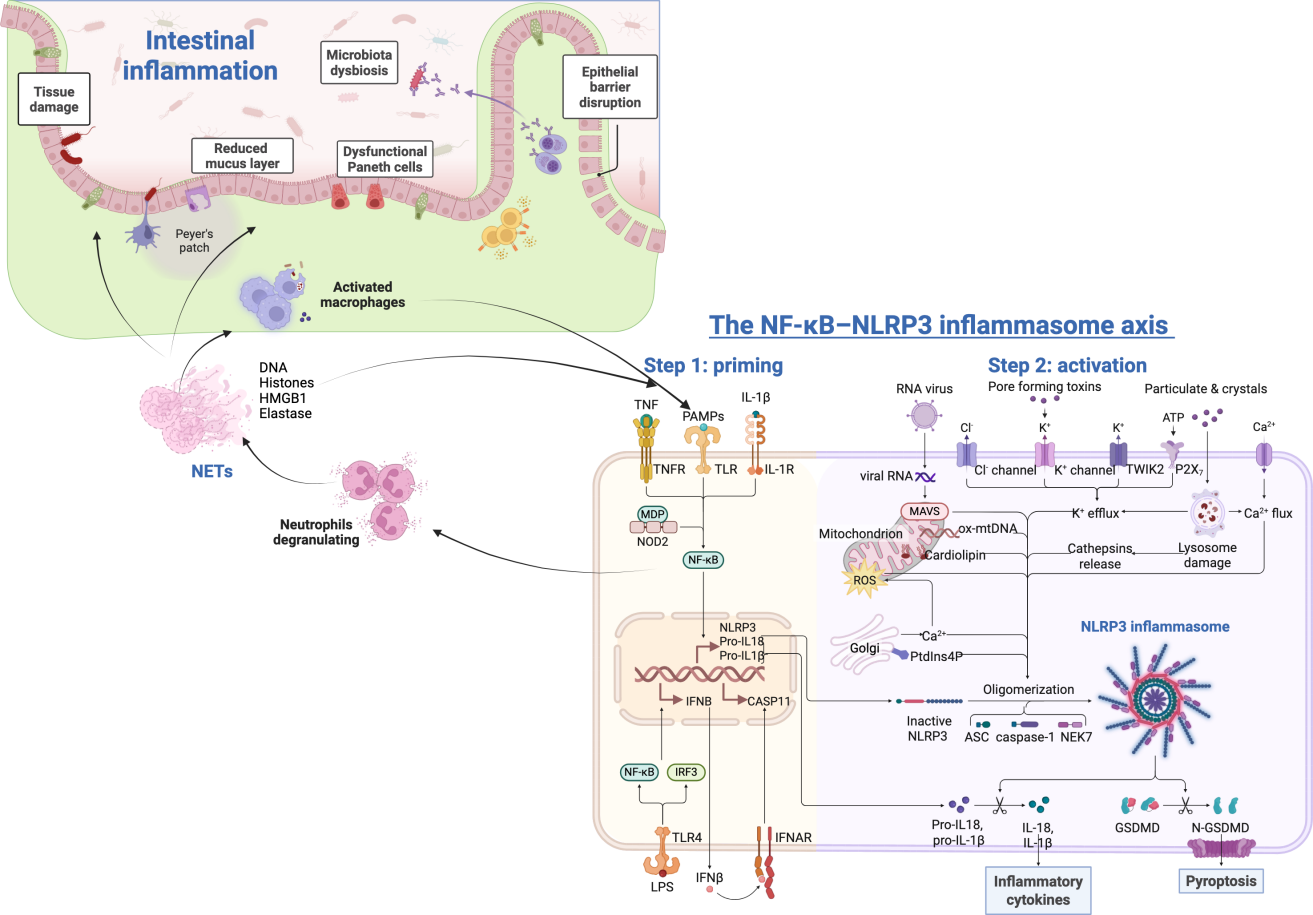
The NF-κB–NLRP3 inflammasome axis and neutrophil extracellular traps (NETs) feedback loop in intestinal inflammation. Microbial triggers (LPS, MDP, PAMPs) activate TLR/PRR signalling, leading to NF-κB-dependent transcriptional priming (Step 1: upregulation of NLRP3, pro-IL-1β, pro-IL-18). Danger signals (ATP, ROS, K^+^ efflux) then trigger NLRP3 inflammasome assembly with ASC oligomerisation and caspase-1 activation (Step 2: activation), resulting in IL-1β/IL-18 maturation and Gasdermin D (GSDMD)-mediated pyroptosis. Simultaneously, NF-κB primes neutrophils for NETosis. Released NETs (containing DNA, histones, HMGB1, elastase) serve as DAMPs that engage TLR2/TLR4/RAGE receptors on epithelial cells and macrophages, reactivating canonical NF-κB signalling and creating a self-amplifying feedback loop that perpetuates intestinal inflammation, epithelial barrier disruption, and tissue damage. This circuit is particularly relevant in NEC, CD, UC, and sepsis (created with BioRender).

Mechanistically, this NF-κB–NETs axis has been demonstrated in several intestinal disease contexts (see **Figure 2**). In CD, NETs were shown to induce intestinal fibrosis through the TLR2/NF-κB pathway, with peptidyl arginine deiminase 4 (PAD4)-dependent NET formation driving early fibrotic remodelling (57), thereby aggravating tissue injury beyond the acute inflammatory phase. Furthermore, restoring NR4A3 function in neutrophils downregulated NF-κB, resulting in decreased NET formation, reduced organ damage, and improved sepsis outcomes, which demonstrates that intervening at the NF-κB node can interrupt the entire NETs cascade (56). Similarly, in rat models of experimental colitis, the administration of hydrogen sulfide (H_2_S) reduced inflammation by inhibiting NF-κB signalling and decreasing NETs formation, leading to fewer colonic lesions (55). Together, these findings establish that NF-κB is not merely associated with NETs, but functions as a druggable node that controls both NET production and the downstream inflammatory response to NET-derived DAMPs.

The NF-κB–NETs axis assumes particular clinical significance in Necrotizing Enterocolitis (NEC), one of the most common causes of morbidity and mortality in preterm infants. In NEC, the combination of mucosal immaturity, aberrant bacterial colonisation, and TLR4 overexpression creates a microenvironment that is highly conducive to excessive neutrophil infiltration and NET release (58). We and others have shown that NETs are abundantly released in NEC intestinal tissue, where they exacerbate epithelial injury and amplify the inflammatory cascade (59). Critically, we demonstrated that enzymatic degradation of extracellular DNA—the structural backbone of NETs—by DNase treatment significantly ameliorated NEC severity in a murine model (60), providing direct evidence that NET-derived DNA is a functional driver of NEC pathology rather than merely a bystander. These findings position NET-targeted strategies as a promising therapeutic approach, complementary to direct NF-κB inhibition, for this devastating neonatal condition.

## 2 NF-κB dysregulation in IBD

Inflammatory bowel diseases (IBD) are chronic conditions characterized by episodes of inflammation in the gastrointestinal tract, being Ulcerative colitis (UC), Crohn’s disease (CD), and Celiac Disease (CeD) the most frequent disorders. Dysregulation of proteins participating in the NF-κB signalling pathway has been shown to contribute to the progression of IBD (11, 20).

Highly regulated NF-κB activity in intestinal epithelial cells (IEC) is essential for normal gut homeostasis and barrier maintenance. Signals from many immune sensors activate NF-κB subunits in the intestine, which maintain an equilibrium between local microbiota and host responses. Genetic association studies of patients with IBD and preclinical mouse models confirm the importance of the NF-κB system in host defence in the gut. (11). Indeed, IBD has been associated with the activation of the NF-κB pathway, leading to an increased transcription of pro-inflammatory mediators that result in diarrhoea, abdominal pain, bleeding, and further extra-intestinal manifestations (61).

Moreover, a study comparing patients of Irritable Bowel Syndrome (IBS) with IBD found a similar pattern of NF-κB p65/p50 expression in IBS patients with predominant diarrhoea and in IBD patients, suggesting possible common pathogenetic pathways in both disorders. Also, an increased expression of NF-κB p50 in IBS patients compared to IBD subjects or controls seems to be an early event in the process of inflammation (62).

A comparative analysis of NF-κB involvement across these conditions (see **Table 1**) reveals an important gradient: while NF-κB activation in IBS appears to be a low-grade, early inflammatory event without overt tissue destruction, it escalates to chronic mucosal inflammation with epithelial barrier disruption in UC and CD, and culminates in the fulminant, often necrotising tissue injury seen in NEC. This gradient correlates with the degree of NF-κB pathway dysregulation—from altered p50/p65 ratios in IBS, through NOD2-driven amplification in CD and persistent canonical activation in UC, to the TLR4-mediated hyperactivation in the immature neonatal gut in NEC. Understanding where each condition falls on this spectrum is essential for calibrating therapeutic NF-κB inhibition, as the optimal degree and target of intervention likely differs substantially between a functional syndrome such as IBS and a life-threatening neonatal emergency such as NEC.

**Table 1:** Comparative summary of NF-κB involvement across intestinal inflammatory disorders.

Feature	IBS	Crohn’s disease (CD)	Ulcerative colitis (UC)	Celiac disease (CeD)	NEC
**Disease type**	Functional (low-grade inflammation)	Chronic inflammatory (transmural)	Chronic inflammatory (mucosal)	Autoimmune (gluten-triggered)	Acute neonatal (necrotising)
**NF-κB activation level**	Low-grade (↑ p50)	Moderate–high (↑↑ p65/p50)	High (↑↑ p65/p50)	Moderate (↑ p50, p65)	Very high (↑↑↑ p65)
**Key NF-κB pathway**	Canonical (p50/p65)	Canonical + NOD2 (MDP sensing)	Canonical + non-canonical	Canonical (TLR/gliadin)	Canonical (TLR4-driven)
**Primary triggers**	Dysbiosis, stress, mast cell mediators	NOD2 mutations, MDP, LPS, dysbiosis	Dysbiosis, epithelial stress	Gliadin peptides, TNFAIP3/REL variants	TLR4 overexpression, aberrant colonisation, mucosal immaturity
**NLRP3 involvement**	Limited evidence	NOD2-dependent priming → NLRP3 assembly, IL-1β	Crypt abscesses, IL-1β/IL-18, pyroptosis	Not well characterised	Heightened pyroptosis in immature epithelium
**NETs involvement**	Minimal	Fibrosis via TLR2/NF-κB/PAD4	Epithelial injury, barrier disruption	Not well characterised	Major driver: DNase treatment ameliorates NEC
**Barrier dysfunction**	NF-κB–MLCK pathway, tight junction loss	Patchy NF-κB hotspots, focal disruption	Continuous mucosal damage, iNOS induction	Villous atrophy, lymphocyte infiltration	Immature barrier, TLR4-driven hyperinflammation
**Therapeutic NF-κB targeting**	Probiotics, polyphenols, low-dose modulators	Anti-TNFα, thalidomide, curcumin	Anti-TNFα, imatinib, baicalin, proteasome inhibitors	Gluten-free diet; NF-κB inhibitors (investigational)	NET-targeted (DNase), IKKβ deletion, REGγ/p65 inhibition
**Tissue outcome**	No macroscopic damage; visceral pain	Transmural ulcers, fistulae, fibrosis	Mucosal ulceration, crypt abscesses, CRC risk	Villous atrophy, crypt hyperplasia, lymphoma risk	Intestinal necrosis, perforation, high mortality

CRC, colorectal cancer; GSDMD, Gasdermin D; MLCK, myosin light chain kinase; MDP, muramyl dipeptide; PAD4, peptidyl arginine deiminase 4.

On the other hand, the increasing prevalence of IBD in industrialized countries suggests that environmental factors are also important determinants of IBD susceptibility and severity. Apart from the microplastics-induced NF-κB activation described above, herbicides like propyzamide have been also reported to activate the AHR–NF-κB–C/EBPβ signalling axis in T cells and dendritic cells, which enhances intestinal inflammation in the small and large intestine (63).

### 2.1 Irritable bowel syndrome

Irritable bowel syndrome (IBS) is the most prevalent functional gastrointestinal disorder, affecting approximately 10–14% of the global population, characterised by chronic abdominal pain, altered bowel habits, and visceral hypersensitivity. Although traditionally classified as ‘functional’, increasing evidence demonstrates that low-grade mucosal inflammation, immune activation, and altered intestinal permeability—particularly in IBS-D and post-infectious IBS (PI-IBS)—take place and are partially mediated in part by NF-κB signalling pathways.

Several lines of evidence implicate NF-κB in IBS pathophysiology. A study comparing NF-κB expression in IBS and IBD patients found similar p65/p50 activation patterns in IBS-D and IBD, with increased p50 expression in IBS compared to controls, suggesting NF-κB activation as an early inflammatory event (62). The NF-κB–MLCK signalling pathway, regulated by TRAF3, contributes to intestinal hyperpermeability in IBS-D (31).

In PI-IBS, the initial infectious insult activates TLR-dependent NF-κB signalling, and failure to resolve this response may lead to persistent low-grade activation, sustained immune cell infiltration (particularly mast cells and T lymphocytes), and chronic visceral hypersensitivity. Mast cell-derived mediators (histamine, tryptase) can activate NF-κB in enteric neurons and epithelial cells, linking immune activation to the visceral pain characteristic of IBS.

IBS patients frequently exhibit altered gut microbiota with reduced diversity, activating TLR4/NF-κB signalling and perpetuating low-grade inflammation. The degree of NF-κB activation in IBS is quantitatively lower than in IBD, consistent with the absence of macroscopic mucosal damage, yet sufficient to drive pain, altered motility, and barrier dysfunction.

Therapeutically, strategies modulating NF-κB activity at a low level—such as specific probiotics, dietary polyphenols, or targeted anti-inflammatory agents—may offer symptomatic relief in IBS without the immunosuppressive risks of aggressive NF-κB inhibition required for IBD. This positioning of IBS on the milder end of the NF-κB activation spectrum underscores the importance of calibrating therapeutic inhibition to the degree of pathway dysregulation.

### 2.2 Crohn’s disease

The NF-κB pathway was already connected to Crohn’s disease (CD) years ago, when a frameshift mutation in NOD2 gene, which encodes a truncated NOD2 protein, was associated with susceptibility to CD. In normal conditions, wild-type NOD2, activates NF-κB, making it responsive to bacterial LPS; however, this induction is impaired when NOD2 is mutated. Consequently, NOD2 implies predisposition to CD, and suggests a link between an innate immune response to bacterial components and development of disease (64).

Later on, other variants of NOD2 gene were identified in CD patients. These variants are thought to be defective in activation of NF-κB and antibacterial defences, because NOD2 acts as an intracellular sensor of bacteria-derived muramyl dipeptide (MDP). Thus, presenting these NOD2 variants would increase susceptibility to bacterial-induced intestinal inflammation, and also increase predisposition to develop CD (10, 65). In addition, it could be demonstrated that NOD2 mutations potentiate NF-κB activity and IL-1β processing in CD clinical samples (65).

Years later, the role of NOD2 within the NF-κB pathway was established: NOD2 activation by the bacterial MDP recruits RIP2, which induces NEMO ubiquitination, activating the IKK signalling complex that then activates the degradation of the inhibitory IκBs and so, releases the NF-κB dimers for the subsequent gene transcription into the nucleus (66).

Despite many patients with CD carry mutations in NOD2, this protein highlights the biphasic nature of the pathology of CD, because NOD2 can both strongly activate and negatively attenuate NF-κB signalling. Different studies implicate NOD2-induced ubiquitination of the NF-κB regulator NEMO as a key mechanism in manipulating the NF-κB signal (66, 67). Specifically, the three major CD-associated NOD2 variants—R702W, G908R, and L1007fsinsC—can be classified along a functional spectrum. L1007fsinsC produces a truncated protein unable to sense MDP, representing a clear loss-of-function mutation with impaired antimicrobial defences and reduced α-defensin secretion by Paneth cells. In contrast, R702W and G908R show reduced MDP sensing (partial loss of function) but can paradoxically potentiate NF-κB activity and IL-1β processing under inflammatory conditions (65), reflecting a gain-of-function component where failure to terminate NF-κB signalling leads to persistent inflammation—explaining the coexistence of impaired mucosal defence and excessive inflammation in CD patients carrying NOD2 variants.

Recently, a heterozygous missense variant in RELA (c.587T>C, p.V196A) that impairs RelA (p65) protein stability, was identified in a CD patient, who presented an atypical Crohn’s-like phenotype (pan-enteric Crohn’s disease (CD), perianal fistulas, chronic mucocutaneous candidiasis, and chronic lymphopenia) (68).

Furthermore, gene polymorphisms in the canonical NF-κB pathway gene, concretely presenting the TLR2 rs3804099 T allele, have been associated with nonresponse to anti-TNFα treatments in CD patients (69).

The inflamed intestine in CD condition usually presents imbalanced apoptosis of enterocytes in the epithelium. Our results show that intestinal samples of CD patients have higher number of GFAP+ enteric glia cells population (EGC), together with increased levels of glial-derived neurotrophic factor (GDNF). Also, pro-inflammatory cytokines induce GDNF expression by GFAP+ enteric glia *in vitro* suggesting that the GFAP+ enteric glia as major cellular source of the upregulated GDNF in the inflamed gut. Therefore, mucosal EGC seems to play a key role in preserving the intestinal epithelium and may contribute to reestablish the integrity of the injured epithelium (70).

Experiments using intestinal epithelial cells of human gut xenografts in SCID mice have demonstrated that systemic LPS and human TNFα or luminal bacteria activate selectively clusters of intestinal epithelial cells in the gut, inducing discrete hotspots of NF-κB activity. This inflammatory hotspots in the normal and inflamed bowel might account for the patchy mucosal lesions typical in CD and thus could have important implications for diagnosis and therapy (71). Importantly, NLRP3 inflammasome activation has been demonstrated in inflamed CD mucous layer, where NOD2-dependent NF-κB priming synergises with bacterial-derived danger signals to drive NLRP3 assembly and IL-1β release, amplifying the local inflammatory response.

The NOD2–NF-κB axis in CD reveals a fundamental paradox that has broader implications for therapeutic targeting of this pathway: NOD2 mutations simultaneously impair the antibacterial NF-κB response (loss of MDP sensing) and potentiate pathological NF-κB activation (enhanced IL-1β processing). This biphasic behaviour suggests that NF-κB dysregulation in CD is not simply a matter of excessive activation, but rather a qualitative shift in signalling output—from protective antimicrobial defence to destructive chronic inflammation. The discovery of patchy NF-κB activation hotspots in the intestinal epithelium (71) supports this view: rather than diffuse hyperactivation, CD appears to involve focal dysregulation driven by local variations in microbial exposure, epithelial barrier integrity, and immune cell composition. The identification of pharmacogenetic determinants of anti-TNFα response (69) further underscores that NF-κB pathway variants not only predispose to disease but also modulate treatment efficacy, making personalised therapeutic approaches based on NF-κB pathway genotyping an important future direction. Complementing these genetic findings, our observation that GFAP+ enteric glia and GDNF are upregulated in inflamed CD tissue (70) adds a neuroimmune dimension: the ENS does not merely respond passively to NF-κB-driven inflammation but actively participates in mucosal defence, positioning enteric glia as potential therapeutic targets for epithelial restitution in CD.

### 2.3 Ulcerative colitis and microscopic colitis

Ulcerative colitis (UC) is a chronic inflammatory disorder affecting the colon mucosa that initially affects the rectum and extends through part or all of the colon in a continuous manner. Over time, it can lead to colorectal cancer (CRC). The NF-κB pathway participates in the pathogenesis of UC by altering cytokine production and further signalling processes in intestinal epithelial cells, lymphocytes, and macrophages in the inflamed intestine (6).

Macrophages activate NF-κB to start inflammation with the production of pro-inflammatory cytokines such as TNFα, IL-1, and IL-6. However, the secretion of other cytokines like IL-12 and IL-23, can ultimately damage the intestinal mucosal tissue, which is characteristic of UC (6). Chronic NF-κB activation has also been observed in the colonic mucosa of UC patients. NF-κB induces the transcription of inducible nitric oxide synthase (iNOS), that usually presents high levels in the colon of patients with collagenous and ulcerative colitis (72). Finally, the NF-κB pathway has been also related to microscopic colitis pathogenesis. The findings suggest that an impairment of the non-canonical NF-κB pathway is involved in the development of MC. Additionally, NLRP3 inflammasome activation has been documented in UC, where it contributes to crypt abscesses and epithelial damage through IL-1β and IL-18-mediated pathways; the NF-κB–NLRP3 axis may thus represent a self-amplifying inflammatory loop in the colonic mucosa of UC patients (73).

Confirming this, treatment with Imatinib decreased NF-κB/p65 activation, and the levels of inflammatory interleukins (IL-23, IL-17, IL-6), which improved UC manifestations in rat models, as evidenced by significantly decreased macroscopic and histological damage in the colon (74).

Notably, the involvement of both canonical (iNOS induction, pro-inflammatory cytokine secretion) and non-canonical (MC pathogenesis) NF-κB pathways in UC-spectrum diseases suggests that the relative contribution of each pathway may vary with disease stage and subtype. This distinction is clinically relevant, as most current therapeutic approaches target the canonical pathway and may therefore be insufficient for conditions in which the non-canonical route plays a predominant role.

### 2.4 Celiac disease

Celiac disease (CeD) is an autoimmune condition caused by intolerance to gluten ingestion that produces chronic intestinal inflammation. The pathogenesis of CeD involves innate and adaptive immunity, primarily mediated by the infiltration of lymphocytes, particularly T cells, into the small intestinal epithelium. The upregulation of the NF-κB pathway and its downstream cytokines, such as IL-8, in the intestinal mucosa suggests the involvement of NF-κB in the development of CeD (6). Gliadin peptides have been reported to activate p50 and p65 subunits and to induce the secretion of pro-inflammatory TNFα and IL-8 in blood monocytes from celiac patients. Also, NF-κB inhibitors reduced both NF-κB DNA binding activity and cytokine production (75).

Furthermore, variants in genes participating in the inflammatory NF-κB pathway, such as in TNFAIP3 (A20, at the protein level) and REL genes, have been identified in celiac patients. A20 shows both ubiquitin ligase and deubiquitinase enzyme activities. A20 negatively regulates NF-κB activation downstream of TLR2 and NOD1/NOD2 (10). Thus, presenting these genetic variants will lead to altered NF-κB signalling and they are associated to a higher celiac disease predisposition. This evidences a role of NF-κB genetic variants for primary heritable predisposing to celiac disease (76).

### 2.5 Necrotizing enterocolitis

NEC represents a paradigmatic example of NF-κB-driven acute intestinal inflammation in the immature gut. Unlike the chronic inflammatory conditions discussed above, NEC occurs in a developmentally unique context: the preterm neonatal intestine is characterised by an immature mucosal barrier, a naïve immune system with limited regulatory T-cell function, and an evolving microbiome that is highly susceptible to dysbiosis (77). This constellation creates a “perfect storm” for NF-κB hyperactivation, as the neonatal intestinal epithelium expresses disproportionately high levels of TLR4 relative to the anti-inflammatory regulators that normally counterbalance TLR signalling in the mature gut.

Overall, the immaturity of the mucosal barrier and the increased expression of Toll-like receptor 4 (TLR4) within the intestinal epithelium result in an intestinal hyperinflammation reaction. NF-κB is persistently active during NEC in rat models, and this overactivity may have a detrimental effect on intestinal tissue. Thus, NF-κB activation in Ly6c^+^ monocytes plays a critical role in promoting intestinal inflammation and therefore in the development of NEC (77). Epithelial cells from NEC patients have an increased innate TLR-4 expression upon LPS stimulation, potentially contributing to NEC development. LPS stimulation resulted in more pronounced NEC-like lesions in NEC organoids, which were aggravated by neutrophils. Thus, neutrophils exacerbate inflammatory lesions in NEC (78).

These findings from our group establish a mechanistic triad in NEC pathogenesis: bacterial LPS stimulates TLR4-overexpressing neonatal enterocytes, which activate NF-κB-dependent cytokine release and recruit neutrophils into the intestinal wall; the infiltrating neutrophils then release NETs that further damage the already fragile epithelial barrier, creating a feed-forward loop of inflammation and tissue destruction. Importantly, this triad—TLR4/NF-κB activation, neutrophil infiltration, and NET-mediated barrier disruption—distinguishes NEC from adult forms of IBD, where the mature epithelium and established immune tolerance provide some degree of resilience against this escalating cascade. Notably, NLRP3 inflammasome activation has also been implicated in NEC, where the immature neonatal intestinal epithelium shows heightened susceptibility to NLRP3-mediated pyroptosis, further exacerbating tissue destruction (79).

The activation of the NF-κB pathway appears to be involved in the pathological intestinal inflammation observed in NEC by disrupting the epithelial barrier in response to REGγ. REGγ activates the degradation of IκBε, which results in the activation of pro-inflammatory NF-κB p65. Clinically, REGγ and p65 expression levels exhibit a positive correlation in NEC specimens. Moreover, pre-treatment of mice with a p65 inhibitor effectively suppressed the expression of inflammatory mediators and alleviated REGγ-mediated NEC development. Thus, a strong NF-κB activity induced by REGγ aggravates NEC development (80).

Additionally, NF-κB is required for NEC-induced monocyte activation, recruitment, and differentiation in neonatal intestines. Furthermore, blocking NF-κB activation in mice by IKKβ deletion ameliorated NEC symptoms and improved survival (81).

In all, the NF-κB pathway is an essential mediator of the inflammation and intestinal injury observed in NEC progression.

## 3 Current therapies for NF-κB inhibition

### 3.1 Approved drugs

There are several therapeutic strategies to inhibit NF-κB signalling clinically already in use. Inhibitors of NF-κB are widely used in various clinical settings for the treatment of tumours, diabetes, and other conditions.

Main NF-κB inhibitory approaches include IKK inhibitors, monoclonal antibodies, proteasome inhibitors, nuclear translocation inhibitors, DNA binding inhibitors, Tyrosine kinase inhibitors, non-coding RNAs, immunotherapy, and CAR-T cells among others (6, 82–84).

One of the most standard drugs blocking NF-κB signalling are NSAIDs, which selectively inhibit IκB to suppress the activation of NF-κB (6), followed by glucocorticoids like dexamethasone, which binds the RelA subunit to inhibit its activity, or by antibiotics like sulfonamides (85). Another example is thalidomide, an immunomodulatory drug approved for multiple myeloma, which suppresses the transcription function downstream of NF-κB (86). Besides, monoclonal antibodies, including anti-PD-L1 (84), anti-IL-1, and anti-TNFα block the binding of ligands and respective receptors to inhibit NF-κB activation (6). There is also a large list of proteasome inhibitors, such as Bortezomib, Carfilzomib, Ixazomib, and Lactacystin, which block IκBs degradation by the proteasome, thereby retaining NF-κB dimers inactive in the cytoplasm, causing apoptosis (6). This makes them good candidates for cancer treatment combinations (87, 88). Additional strategies include the IκBα super-repressor, or anti-miR oligonucleotides, which can be used to inhibit miRNAs that promote the NF-κB signalling pathway (6).

### 3.2 Natural compounds

On the other hand, using natural compounds to reduce pro-inflammatory NF-κB signalling represents an alternative with fewer adverse effects than other medicines on the market. Diverse polyphenols contained in fruits and vegetables can downregulate inflammatory NF-κB and so, block the synthesis of cytokines and the activation of immune cells. The list includes curcumin, Epigallocatechin gallate (EGCG found in green tea), resveratrol, and quercetin. Evidence from preclinical studies indicate a reduction in intestinal inflammation by these compounds (89, 90).

Resveratrol, contained in grapes, is one of the most widely used polyphenols to inhibit NF-κB. Resveratrol has been shown to maintain intestinal barrier function by reducing the expression of apoptotic proteins, enhancing intestinal tight junction proteins, and inhibiting the inflammatory response mediated by the TLR4/MyD88/NF-κB signalling pathway, thereby alleviating LPS-induced septic intestinal injury (91).

Berberine is an isoquinoline alkaloid found in a variety of plant species that has anti-inflammatory properties. For many years, berberine has been used to treat intestinal inflammation and infection. Berberine has been reported to reduce LPS-induced intestinal damage by inhibiting NF-κB (92).

Curcumin, a well-known NF-κB inhibitor reported to block Nod2 signalling (93), has been shown to induce clinical remission in UC patients and reduced clinical relapse in quiescent UC patients. Since curcumin is a nontoxic, inexpensive, and easily available natural polyphenol; it has an enormous potential in the treatment of patients with moderate UC (94).

Further natural compounds that have emerged as a good option for managing UC manifestations, like improving intestinal barrier, restoring the mucosa and reducing inflammation) are: Baicalin (95), Paeonol (96), and Parthenolide (97, 98). Besides, Micheliolide, a new sesquiterpene lactone, inhibits intestinal inflammation and colitis-associated cancer (99).

Also, fungal metabolites (such as galiellalactone, and dehydrocurvularin), show anti-inflammatory effects in animal models of IBD (100).

Finally, a large study assessing fifty-six phytochemicals (curcumin, resveratrol, kaempferol, sesamol, pinocembrin, astragalin, oxyberberine, berberine hydrochloride, botulin, taxifolin, naringin, thymol, isobavachalcone, lancemaside A, aesculin, tetrandrine, Ginsenoside Rk3, mangiferin, diosgenin, theanine, tryptanthrin, lycopene, gyngerol, alantolactone, mangostin, ophiopogonin D, fisetin, sinomenine, piperine, oxymatrine, euphol, artesunate, galangin, and nobiletin) described a decrease in NF-κB pro-inflammatory cytokines (TNF-α, IL-1β, IL-6, IFN-γ), and cyclooxygenase-2 (COX-2), followed by an increase in keepers of the epithelial barrier (occludin, claudin-1, zonula occludens-1), and IL-10 expression levels. Moreover, phytochemicals improved weight loss, stool consistency, and rectal bleeding in IBD (61).

### 3.3 Probiotics as NF-κB modulators

Given the central role of microbiota dysbiosis in NF-κB-mediated intestinal inflammation, probiotics represent a rational therapeutic approach. *Lactobacillus rhamnosus* GG inhibits NF-κB by stabilising IκBα. *Bifidobacterium infantis* 35624 induces regulatory T cells and suppresses NF-κB-dependent cytokine production in IBS clinical trials. *Saccharomyces boulardii* blocks IKK/IκB signalling and reduces CD relapse rates. VSL#3 maintains remission in UC and pouchitis with reduced mucosal NF-κB p65 translocation. Mechanistically, probiotics suppress NF-κB through competitive exclusion of pathogens, SCFA production (especially butyrate, which directly inhibits NF-κB nuclear translocation), barrier enhancement, and IL-10 induction. Fecal Microbiota Transplantation (FMT) also attenuates NF-κB signalling (29), with emerging UC evidence. While probiotics offer an attractive safety profile, efficacy varies by strain, dose, and condition; standardised RCTs with NF-κB activity as a mechanistic endpoint are needed.

Among natural compounds, curcumin has a favourable safety profile in UC trials but limited bioavailability. Resveratrol and berberine show preclinical promise but limited human data, with potential CYP3A4/CYP2D6 interactions warranting caution. EGCG and quercetin are well-tolerated at dietary doses but may cause hepatotoxicity at high supplemental doses. A general concern is that systemic NF-κB inhibition may impair host defence (immune surveillance, antimicrobial responses, tumour suppression), underscoring the need for cell-type-specific or tissue-targeted approaches.

Anti-TNFα antibodies (infliximab, adalimumab) are the most established biologics for moderate-to-severe IBD but carry risks of serious infections (including TB reactivation), infusion reactions, immunogenicity, and modestly increased lymphoma risk with prolonged use. Concomitant immunosuppressants reduce immunogenicity but further increase infection risk. Imatinib shows experimental UC efficacy but clinical IBD experience is limited, with oedema, nausea, and hepatotoxicity as concerns.

While NF-κB inhibition offers substantial therapeutic promise, the clinical effectiveness, safety profile, adverse effects, and drug interactions of the discussed approaches require consideration. NSAIDs carry gastrointestinal toxicity risks (ulceration, bleeding) and may paradoxically exacerbate IBD; their use requires careful monitoring. Glucocorticoids are effective for acute flares but unsuitable for maintenance due to osteoporosis, adrenal suppression, metabolic syndrome, and infection susceptibility. Thalidomide is effective in refractory CD but strictly contraindicated in pregnancy (teratogenicity) and limited by peripheral neuropathy, sedation, and thromboembolism. Proteasome inhibitors (e.g., bortezomib) show preclinical promise in UC but are limited by neuropathy, thrombocytopenia, and GI side effects.

In short, there are several approaches available to target NF-κB (see **Table 2**), from approved drugs to natural compounds, and most of them have already been proved to ameliorate intestinal inflammation symptoms.

**Table 2:** NF-κB inhibitory compounds and drugs with demonstrated effects on intestinal inflammation.

Compound	Mechanism of NF-κB inhibition	Improved disorder	References
Approved drugs			
NSAIDs	IκB inhibition	Various inflammatory conditions	(6)
Dexamethasone	RelA subunit binding	Various inflammatory conditions	(6)
Sulfonamides	NF-κB suppression	Bacterial infections with inflammation	(85)
Thalidomide	Downstream transcription suppression	Multiple myeloma Intestinal inflammation	(86, 101, 102)
Bortezomib	Proteasome inhibition (IκB stabilisation)	Multiple myeloma, cancer UC, lymphomagenesis in coeliac disease	(87, 88, 103–105)
Anti-TNFα antibodies	Ligand–receptor binding block	IBD (CD, UC)	(6)
Imatinib	Tyrosine kinase inhibition, p65 suppression	Ulcerative colitis (rat model)	(74)
Natural compounds			
Baicalin	IKK/IκB/NF-κB pathway inhibition	Ulcerative colitis	(95)
Berberine	NF-κB inhibition, LPS-induced damage reduction	Intestinal inflammation	(92)
Curcumin	Nod2 signalling block	IBD, ulcerative colitis	(61, 94)
Resveratrol	TLR4/MyD88/NF-κB pathway inhibition	Intestinal barrier, septic injury	(91)
Quercetin	TLR4/NF-κB downregulation	Intestinal inflammation	(90)
EGCG (green tea)	NF-κB downregulation	Intestinal inflammation	(89)
Parthenolide	NF-κB inhibition, barrier restoration	Ulcerative colitis, sepsis	(97, 98)
Paeonol	PPAR-γ modulation, NF-κB suppression	Ulcerative colitis	(96)
Micheliolide	NF-κB inhibition	Colitis-associated cancer	(99)
Fungal metabolites	NF-κB suppression	IBD (mouse model)	(100)

## 4 Open questions and future directions

Despite the substantial body of evidence linking NF-κB dysregulation to intestinal inflammatory diseases, several important questions remain unresolved and warrant dedicated investigation.

A central challenge concerns the translational gap between preclinical evidence and clinical application. Most data on NF-κB inhibitors in intestinal inflammation derive from animal models and *in vitro* systems; well-designed randomised controlled trials in humans are largely lacking, particularly for natural compounds such as curcumin, resveratrol, and berberine. Moreover, the pleiotropic nature of NF-κB signalling raises concerns about the specificity and long-term safety of its therapeutic inhibition, since NF-κB is also essential for normal immune surveillance, tissue repair, and tumour suppression. The development of cell-type-specific or tissue-targeted NF-κB modulators could overcome the limitations of systemic inhibition and open new therapeutic avenues for both IBD and NEC.

Several disease-specific knowledge gaps also remain. The precise mechanisms by which NETs activate NF-κB in the neonatal intestine, and how this intersects with the immature mucosal barrier and TLR4 overexpression in NEC, require further elucidation; this is particularly relevant given the limited therapeutic options available for preterm neonates. Similarly, while the gut–brain axis and microplastics-mediated dysbiosis represent rapidly emerging fields, the relative contribution of NF-κB to these processes compared to other inflammatory pathways (e.g., JAK-STAT, MAPK) remains to be defined. The NF-κB-specific literature in IBS is also largely preclinical, and its role relative to mast cell or serotonergic pathways in visceral hypersensitivity requires clarification.

Methodological limitations of this review should also be acknowledged. The studies discussed span different species, cell types, and experimental conditions, which limits direct comparability. Long-term human exposure data for microplastics and their intestinal inflammatory effects are still lacking, and causality remains difficult to establish from the available observational and animal data.

Future research should prioritise randomised controlled trials of NF-κB inhibitors in human IBD and NEC cohorts, the development of biomarkers for NF-κB pathway activity to guide personalised therapeutic approaches, and the investigation of combination strategies—such as probiotics with natural compounds or NET-targeting agents with conventional anti-inflammatory therapies—to maximise efficacy while minimising systemic immunosuppression.

## 5 Concluding remarks

In conclusion, this review establishes the NF-κB pathway as a central integrator of intestinal inflammation, linking microbiota dysbiosis, immune activation, neutrophil extracellular trap formation, and gut–brain axis signalling into a unified pathological framework. Three principal insights emerge from the evidence presented. First, the bidirectional amplification loop between NF-κB and NETs represents a particularly important and therapeutically actionable mechanism: NF-κB-dependent priming drives NETosis, while NET-derived DAMPs reactivate NF-κB in surrounding epithelial cells and macrophages, creating a self-sustaining inflammatory circuit that is especially destructive in the immature neonatal intestine. Second, NF-κB activation follows a clinically meaningful gradient across intestinal disorders—from low-grade pathway engagement in IBS, through chronic mucosal dysregulation in Crohn’s disease and ulcerative colitis, to fulminant TLR4-mediated hyperactivation in NEC—suggesting that therapeutic NF-κB inhibition must be calibrated to the degree and context of pathway dysregulation. Third, emerging environmental threats, particularly microplastics, converge on the same NF-κB/NLRP3 axis through both direct epithelial interaction and microbiota-mediated mechanisms, underscoring the pathway’s role as a common denominator of diverse intestinal insults.

Given the availability of approved drugs, natural polyphenols, and probiotic-based strategies that target NF-κB at different levels of the signalling cascade, a combinatorial and disease-stage-adapted approach appears most promising.

Further research into the molecular mechanisms and regulatory networks of NF-κB in intestinal inflammation, particularly the development of cell-type-specific modulators and NET-targeted therapies, should provide guidance for novel immunotherapeutic strategies that can be tailored to the specific position of each disorder along the NF-κB activation spectrum.
